# UV damage mapping reveals different impacts of yeast XPD mutations on global genomic and transcription-coupled DNA repair

**DOI:** 10.1016/j.jbc.2026.113483

**Published:** 2026-08-25

**Authors:** Allyson Hoag, Alan E. Tomkinson, Peng Mao

**Affiliations:** 1 University of New Mexico Comprehensive Cancer Center, Albuquerque, New Mexico, USA; 2 Department of Internal Medicine, University of New Mexico School of Medicine, Albuquerque, New Mexico, USA; 3 Stony Brook Cancer Center, Stony Brook University, Stony Brook, New York, USA; 4 Department of Pathology, Stony Brook University School of Medicine, Stony Brook, New York, USA

**Keywords:** DNA adducts, ERCC2, genome stability, xeroderma pigmentosum group D, damage mapping

## Abstract

Two subpathways of nucleotide excision repair (NER), global genomic (GG-NER) and transcription-coupled NER (TC-NER), remove bulky DNA lesions such as cyclobutane pyrimidine dimers (CPDs). Xeroderma pigmentosum protein D (XPD) is a DNA helicase subunit within the transcription factor IIH complex that is important for DNA unwinding and damage verification during NER. Germline mutations in XPD can not only cause the cancer-prone disease xeroderma pigmentosum (XP), but also a combination of XP with Cockayne Syndrome (CS), a syndrome characterized by neurodegeneration and premature aging. While XP and XP/CS mutations in XPD disrupt NER, their specific effects on the NER subpathways are less well understood. In this study, we introduced two pairs of XP and XP/CS mutations into the yeast *RAD3* gene, a homolog of human *XPD*. Our data indicates that yeast XP/CS mutants are more sensitive to UV radiation and have reduced NER capacity relative to the XP mutants. Using a damage mapping method named CPD sequencing (CPD-seq 2.0), we found that the XP/CS mutations abrogated repair by both GG-NER and TC-NER, while XP mutations impaired GG-NER but retained significant TC-NER activity. Consistent with these results, XPD mutant human cell lines established from individuals with XP/CS were sensitivity to Illudin S, an agent that induces damage repaired by TC-NER. Thus, we conclude that XP/CS mutations not only cause a more severe overall NER defect than XP mutations, but specifically disrupt the TC-NER subpathway, with this defect likely underlying the Cockayne Syndrome symptoms.

NER consists of two intertwined subpathways, global genomic NER (GG-NER) and transcription-coupled NER (TC-NER) (1, 2). Notably, inherited defects in GG-NER and TC-NER have been identified as the causative factors in xeroderma pigmentosum (XP) and Cockayne syndrome (CS), respectively (3, 4). While sensitivity to sunlight is a common characteristic of both diseases, individuals with XP have a high incidence of skin cancers whereas individuals with CS exhibit neurodegeneration, growth and developmental delays, and premature aging (3, 5, 6). The main differences between GG-NER and TC-NER occur in the first step in the repair pathway, DNA damage recognition. GG-NER utilizes damage surveillance proteins, UV-DDB and XPC, to recognize helix-distorting damage, such as UV-induced cyclobutane pyrimidine dimers (CPDs), across the whole genome. XPC senses the thermodynamically destabilized DNA structure rather than a specific type of DNA damage, enabling GG-NER to repair a broad range of DNA lesions (7, 8, 9). In contrast, TC-NER relies on elongating RNA Polymerase II (RNAP II) to identify damage in actively transcribed genes. RNAP II is strongly stalled by CPDs and other bulky DNA adducts, with the stalled RNAP II serving as the signal that recruits Cockayne Syndrome protein B (CSB) to initiate TC-NER (10, 11). While CSB is a SWI/SNF-type ATPase that normally promotes RNAP II forward translocation, upon transcription stalling, CSB quickly moves to the stalled RNAP II and recruits TC-NER factors, such as CSA and UVSSA, to initiate DNA repair (12). Notably, TC-NER activity is restricted to the transcribed strand (TS, also known as template strand). In contrast, GG-NER repairs both strands of duplex DNA and is not restricted to transcribed regions of the genome (13, 14). After damage recognition, the two subpathways converge with the recruitment of Transcription Factor IIH (TFIIH) to facilitate DNA unwinding and pre-incision complex assembly (4, 15, 16). Thus, the lower efficiency of GG-NER compared with TC-NER presumably reflects differences in the efficiency of damage repair throughout the genome *versus* within transcribed regions.

TFIIH is a 10-subunit protein complex that plays important roles in both transcription and nucleotide excision repair (NER) (16, 17, 18, 19). TFIIH consists of a 7-subunit core, including two ATP-dependent DNA translocases and helicases – XPB and XPD, and a 3-subunit CDK-activating kinase module (20). During gene transcription, TFIIH’s XPB subunit opens the promoter DNA, allowing the single-stranded template DNA to engage the RNAP II active site for RNA synthesis. Additionally, the kinase module phosphorylates serine residues within the C-terminal domain of RNAPII to regulate promoter escape, elongation, and mRNA processing (17, 21, 22). In NER, TFIIH mainly functions to unwind the two strands at the damage site after the removal of the CDK-activating kinase module by XPA (21, 23). In GG-NER, XPC interacts with the p62 subunit of TFIIH to recruit the TFIIH complex to the damage sites, with XPC holding the 3′ side and XPB binding to the 5′ side of the DNA adduct. XPB then initiates DNA unwinding with XPD presumed to complete unwinding, along with lesion verification, to generate a pre-incision bubble of ∼30 nucleotides (4, 7, 24, 25, 26). The damaged strand is subsequently cleaved by the ERCC1-XPF and XPG endonucleases. Although it is generally assumed that TFIIH plays a similar role in TC-NER, how TFIIH functions in TC-NER is much less understood (27). Notably, TC-NER does not require XPC and the two DNA strands around the damage site are already unwound in the RNAP II transcription bubble of 8 to 22 nucleotides, suggesting that less DNA unwinding by TFIIH is required during TC-NER to generate the pre-incision bubble (28, 29).

An intriguing feature of the genes encoding the XPD and XPB subunits of TFIIH, as well as XPG endonuclease, is that in addition to the identification of mutations that cause XP, there are examples of individuals with mutations in these genes that have the symptoms of both XP and CS (3, 30, 31). The impact of these mutations that cause a combination of XP and CS (*i.e.*, XP/CS) on GG- and TC-NER has not been rigorously examined. This prompted us to investigate mutations in *XPD* that either causes XP or XP/CS. The XPD subunit of the essential transcription initiation and NER factor TFIIH contains two helicase domains, an arch domain, an iron-sulfur ring (4FeS), a p44 interacting region, and seven helicase motifs (Fig. 1*A*) (32, 33, 34). In addition to mutations causing XP or XP/CS, additional mutations in *XPD* that cause a third genetic disorder, trichothiodystrophy (TTD) have been identified. Based on their location, the amino acid changes causing TTD are predicted to subtly disrupt the assembly and/or stability of the TFIIH complex causing transcriptional defects (3, 35). In contrast, amino acid changes resulting in XP and XP/CS are enriched in the two XPD helicase domains with biochemical studies indicating that they impair XPD helicase activity (3, 32). Remarkably, some of the amino acid changes that cause XP/CS occur very close to amino acid changes that only cause XP. Table S1 lists the XP and XP/CS mutations in XPD protein and in which domain they are present. For example, the most frequently observed mutation in *XPD*, accounting for ∼75% of detected XPD mutations (36), results in replacement of Arg683, near helicase motif VI, with Trp (R683W). This mutation causes XP (Fig. 1*A*), whereas the change of Gly675 to Arg (G675R) causes XP/CS. Similarly, an amino acid change, Arg601to Leu (R601L), causing XP is immediately adjacent to an amino change, Gly602 to Asp (G602D) that causes XP/CS (Fig. 1*A*) (3, 36). However, it remains unclear why closely spaced mutations in XPD protein cause distinct clinical phenotypes such as XP and XP/CS.

**Figure 1:**
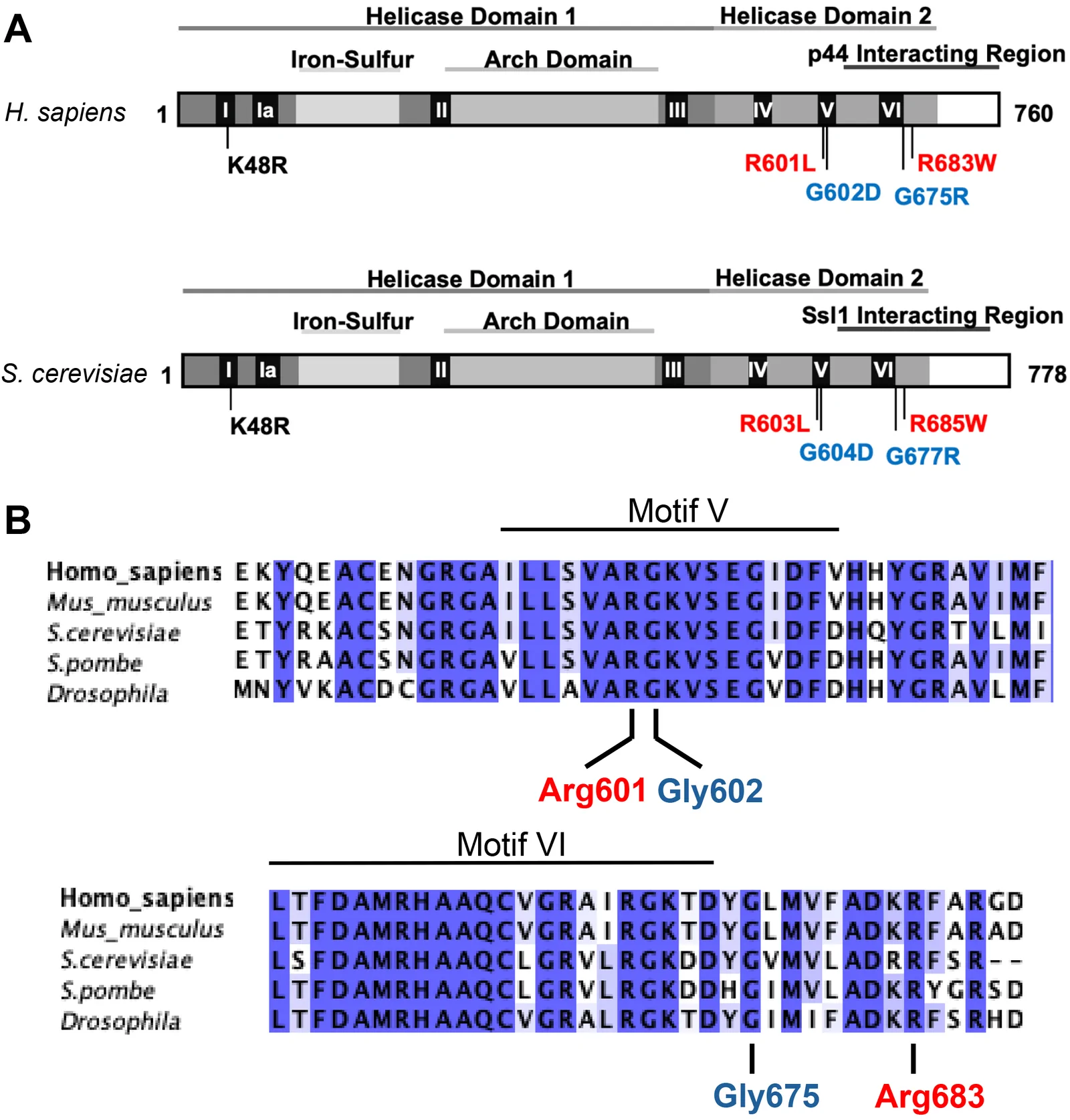
**Conservation of XPD across species.***A,* structure of human XPD showing the locations of each of its domains and motifs, including helicase domain I, helicase domain 2, iron sulfur (4Fe-4S) domain, arch domain, p44 interacting region, and six helicase motifs. Also shown are XP mutations (R683W, R601L) in *red* and XP/CS mutations (G675R, G602D) mutations in *blue*, and the positions of the equivalent mutations in *S. cerevisiae*. *B,* alignment of human XPD with several species for homology near helicase motif V and VI. Shades of *blue* show the amount homology, *darker blue* indicating that residue is conserved across all species. The mutations chosen in our study are indicated.

Previous studies suggested that the CS phenotype in XP/CS cases may arise from persistent TFIIH binding to DNA damage and long-lasting NER intermediate (30, 37). While the detailed mechanism by which XPD mutations disrupt proper TFIIH dissociation remains unclear, TFIIH stalled on DNA damage likely leads to severe reduction in both GG- and TC-NER, because the repair intermediate cannot be further processed in either subpathway. On the other hand, XP is generally considered a GG-NER disorder (1). Hence, the XP-causing mutations in XPD are expected to selectively block GG-NER but impose a lesser severe effect on TC-NER. A published study showed reduction of overall NER activity using an *in vitro* repair system containing XP- or XP/CS-causing XPD protein, but the data did not distinguish between GG- and TC-NER (38). To address deficiency in GG- or TC-NER associated with each XPD mutation, a strand-specific DNA repair assay is needed. In this study, we used yeast as the model organism. Although yeast does not exhibit XP or XP/CS phenotypes as humans, the fundamental NER mechanism, including both GG- and TC-NER, is highly conserved between yeast and humans, making yeast an ideal model organism to understand the impacts of specific XPD mutations on the two NER subpathways. Notably, yeast Rad3 is highly homologous to human XPD (Fig. 1*B*), enabling us to identify amino acids in Rad3 corresponding to XPD amino acids associated with to XP or XP/CS (34). Using CRISPR-Cas9, we introduced point mutations to mimic amino acids that cause XP or XP/CS in the yeast Rad3. We then utilized CPD-seq 2.0, a high-throughput UV damage mapping method that measures repair of UV-induced CPD lesions on the TS and non-transcribed strand (NTS) of yeast genes (14, 39, 40). Our data reveals that the mutant *rad3* alleles corresponding to XP/CS mutations abrogate repair by both GG-NER and TC-NER, whereas the mutant *rad3* alleles corresponding to XP mutations retain TC-NER activity. In accord with these results suggesting that XP/CS cells have a defect in TC-NER, XPD mutant human cells from individuals with XP/CS were sensitive to Illudin S (41), an agent that causes damage repaired by TC-NER.

## Results

### Key XPD amino acids implicated in either XP or XP/CS are conserved in yeast Rad3

Eukaryotic homologs of human XPD exhibit a high degree of sequence similarity, reflecting the functional conservation of this key protein in transcription initiation and NER (34, 41). Notably, XPD amino acids identified as the mutation sites that cause XP (R601L, R683W) or XP/CS (G602D, G675R) are conserved in the *Saccharomyces cerevisiae* XPD homolog, Rad3 (Fig. 1*B*). The significant similarity between the protein sequences of *S. cerevisiae* Rad3 and human XPD provided an opportunity to explore the roles of mutant versions of XPD in NER using yeast as a model organism. We introduced four point mutations into the yeast *RAD3* gene using CRISPR-Cas9 (42) to mimic the mutations associated with XP (R685W, R603L) and XP/CS (G677R, G604D) (Fig. 1*A*). An additional mutation, Rad3 Lys48 to Arg (K48R), which results in the key lysine residue in helicase motif I with an Arg residue, was also introduced to generate a yeast strain expressing a mutant version of Rad3 without NER activity (Fig. 1*A*) (14). The mutation in each strain was confirmed by Sanger sequencing. Additionally, immunoblotting showed that the mutant versions of Rad3 protein were present at steady state levels comparable to WT Rad3 in all the strains (Fig. S1), indicating that these mutations do not affect Rad3 protein synthesis and stability.

### XP and XP/CS mutations in *RAD3* differentially affect UV sensitivity and CPD repair

To determine the effects of mutant versions of Rad3 that mimic XPD proteins expressed in either XP or XP/CS on NER, we first exposed the WT and mutant strains to various doses of UVC using a spotting assay to test their sensitivity (Fig. 2*A*). As expected, the WT strain was resistant to a wide range of UV doses whereas the strain expressing the helicase dead version of Rad3 (K48R) was hypersensitive to UV light, consistent with the published data (14). Interestingly, the two strains expressing mutant versions of Rad3 associated with XP/CS (G677R, G604D) mutants exhibited similar sensitivity to the K48R strain, whereas the strains expressing mutant versions of Rad3 associated with XP (R685W, R603L) exhibited UV sensitivity that was intermediate between the WT strain and the helicase dead mutant. Thus, expression of mutant versions of Rad3 causing XP/CS confers significantly more sensitivity to UV light compared with expression of mutant versions of Rad3 causing XP.

**Figure 2:**
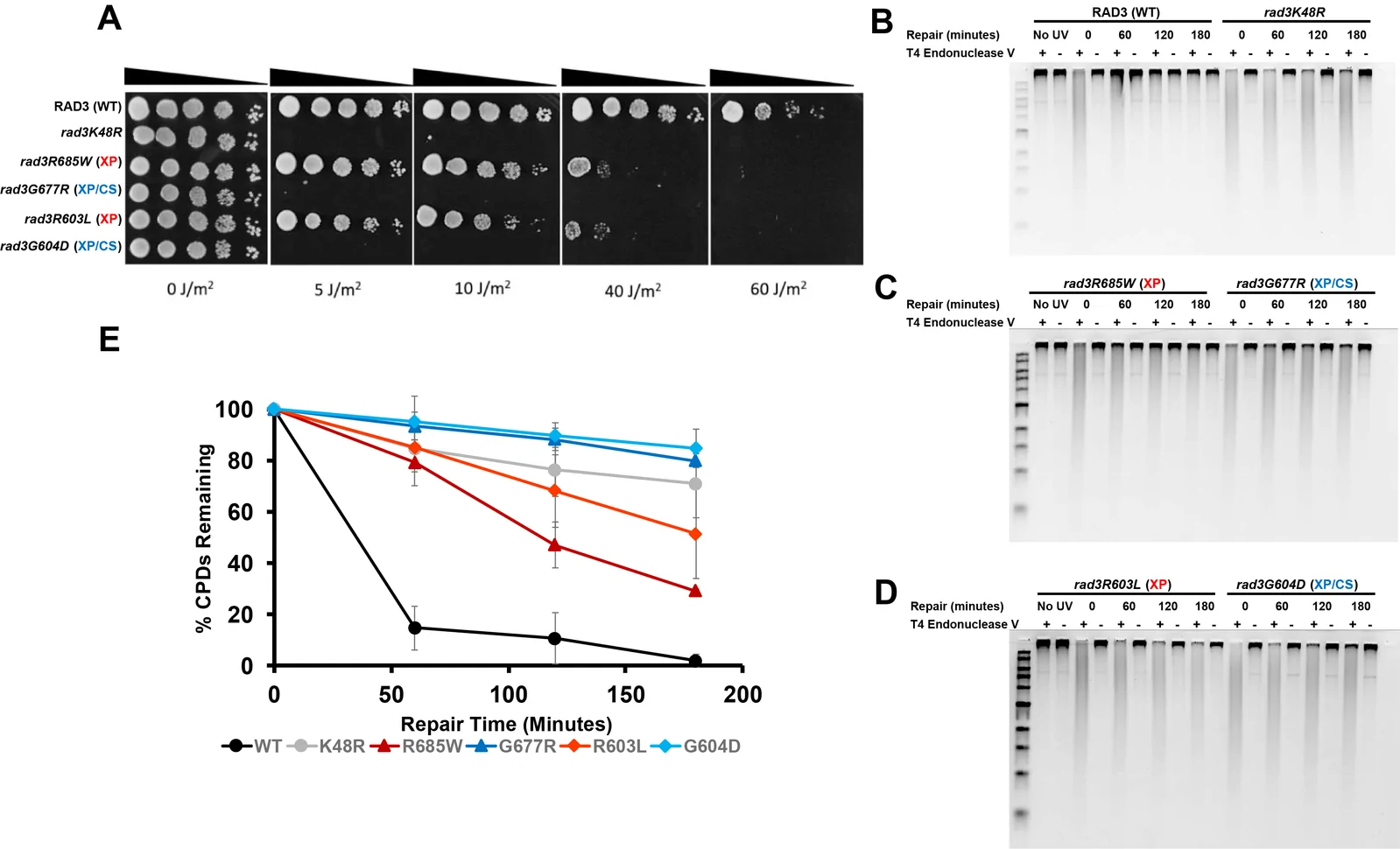
***rad3* XP and XP/CS mutations are differentially sensitive to UV irradiation.***A,* yeast strains were grown at 30 °C to mid-log phase, 10-fold serially diluted, spotted onto YPD plates, and exposed to the indicated doses of UVC *light*. Images were collected after growth at 30 °C for 2 to 3 days in the *dark*. Spot tests were performed in biological triplicate. *B–D,* alkaline gels of the overall levels of UV-induced CPD lesions remaining in WT and *rad3* mutants after repair (43). Cells were grown at 30 °C and damage was induced with 50 J/m^2^ UVC light. Genomic DNA was isolated at the specified timepoints, treated with or without T4 Endonuclease V enzyme, and the samples were electrophoresed on denaturing alkaline gels. Gels were scanned using the Typhoon FLA 7000 imager. *E,* quantification of CPDs remaining in WT and *rad3* mutants based on alkaline gel analysis using *ImageQuant*. The results are plotted as the mean and the standard deviation of 3 to 4 independent replicates. CPD, cyclobutane pyrimidine dimer.

To determine whether differences in cell survival following UV exposure correlated with DNA repair capacity, we measured the overall repair of CPD lesions at various times after UV irradiation using T4 endonuclease V (T4 endo V), which specifically cleaves CPD damage, generating DNA nicks that can be detected by denaturing alkaline gel electrophoresis (43). A representative gel for each strain showing the extent of CPD removal 3 h post irradiation is shown in Figures 2, *B*–*D* and S2, *A*–*E*. Immediately after UV treatment (0 h of post irradiation incubation), all yeast strains had significant and comparable levels of UV damage. Robust repair was evident in the WT cells after 60 min as indicated by the reduction of smearing. At 120 and 180 min, almost all the DNA in WT was shifted to a high molecular weight that corresponds to intact genomic DNA (gDNA). In contrast, in the *rad3* helicase dead (K48R) and XP/CS mutants (G677R, G604D), smearing was retained even at late times; indicating that very little damage was repaired. The *rad3* XP mutants (R685W, R603L) again had an intermediate phenotype, with a slower and incomplete recovery of intact gDNA compared to WT cells, but more recovery than the XP/CS strains. We calculated the percentage of unrepaired CPDs in each strain by quantifying the average CPDs per kilobase (43) over the time course of repair and normalizing to the 0 h timepoint (Fig. 2*E*). As the level of remaining CPDs is inversely proportional to the repair efficiency, the quantification data revealed fast overall repair in the WT strain, with only a very low percentage of CPDs remaining 60 min post irradiation. In contrast, more than 80% of the CPDs remained 180 min after irradiation in the *rad3* XP/CS (G677R, G604D) and helicase dead (K48R) strains, confirming that very little repair occurred in these strains. While both *rad3* XP mutants (R685W, R603L) showed a higher level of CPD removal compared to the XP/CS mutants, these strains were less efficient than the WT in CPD repair. After 180 min of repair incubation, between 40% (R685W) to 60% (R603L) of the initial CPD lesions remained in the two XP strains. The slightly reduced repair in the R603L strain compared with the R683W strain was observed in multiple experiments and is consistent with the moderately elevated UV sensitivity of the R603L strain compared with the R685W strain in the survival assay (Fig. 2*A*).

### Genome-wide mapping of CPDs using CPD-seq 2.0

While the UV survival and CPD repair assays revealed mild and severe repair defects in the XP and XP/CS mutants, respectively, they did not provide insights into the contribution of the GG-NER and TC-NER subpathways in the mutant strains. To discern the activities of the two subpathways, we utilized a high-throughput CPD mapping method CPD-seq 2.0 (39, 40, 44) that enables precise location mapping of CPDs in gDNA to track CPD repair on the TS and NTS (Fig. 3*A*). We prepared CPD sequencing libraries from the WT, K48R, XP (R685W and R603L), and XP/CS mutants (G677R and G604D) before UV treatment (No UV), immediately after UV treatment (0 h), and after 1- or 2-h incubation post irradiation. CPD formation is known to target two adjacent pyrimidines in DNA. Importantly, analysis of the two nucleotides upstream of the 5′ end of each sequencing read revealed strong sequence bias for di-pyrimidines, particularly TT, in UV-treated cells, but not in the unirradiated control (Fig. 3*B*), confirming the mapping specificity for UV damage sites. Additionally, we visualized raw CPD reads in transcribed genes. As shown in two representative genes with opposite transcription directions on chromosome II, CPD reads are roughly even between the TS and NTS at 0 h. However, the number of CPD reads decreased significantly after 1 h repair in WT cells, particularly on the TS of both genes (Fig. 3*C*). The preferential removal of CPDs from the TS is consistent with strand-biased repair by TC-NER. In contrast, there was little repair on either the NTS or TS even 2 h after irradiation in the K48R strain (Fig. S4), indicating that, as expected, loss of Rad3 helicase activity is detrimental for both GG-NER and TC-NER.

**Figure 3:**
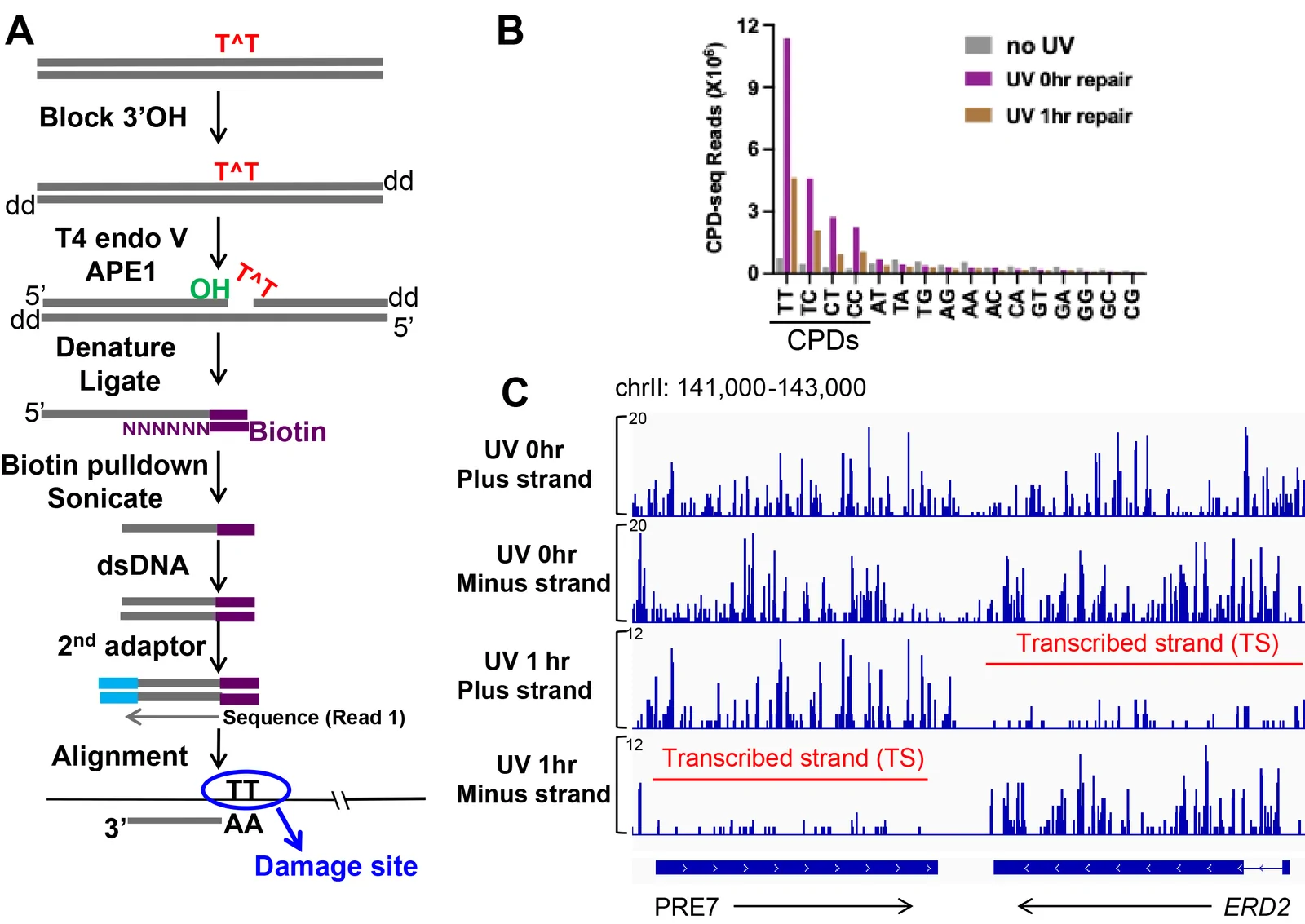
**CPD-seq 2.0 method for mapping UV damage and repair.***A,* CPD-seq 2.0 schematic of sequencing library preparation steps. T^^^T represents most common UV damage (CPD) (39). “OH” indicated a free 3′OH. “dd” indicated dideoxy. *Purple* adapters are the Illumina sequencing adapter with a biotin label and *blue* is the secondary adapter before PCR amplification. *B,* analysis of dinucleotide sequences associated with the 5′ end of CPD-seq sequencing reads. Enrichment of di-pyrimidine sequences (*e.g.*, TT, TC, CT, CC), which are the known CPD-forming sites, is found after UV treatment when compared to No UV. *C,* distribution of CPD reads at 0 h and 1 h of repair for *PRE7* and *ERD2* genes in WT cells. The *red line* indicates the TS, and the y axis depicts the number of CPD-seq reads. Gene coordinates and image drawn using the Integrative Genomics Viewer. CPD, cyclobutane pyrimidine dimer; TS, transcribed strand.

### XP and XP/CS mutations have different effects on GG-NER and TC-NER

The CPD sequencing data provided the opportunity to interrogate GG-NER and TC-NER defects in the XP and XP/CS mutant strains. To analyze the two NER subpathways, we focused on RNAP II-transcribed genes (∼5000) in the yeast genome. Because genes have different sizes, we split the coding region of each gene (*e.g.*, from transcription start site [TSS] to transcription termination site [TTS]) into six equal-sized bins and counted CPD reads in each bin. The remaining CPD count after repair was normalized to the initial count at 0 h to calculate the fraction of remaining CPDs in each bin, which is inversely correlated with repair efficiency (*e.g.*, high fraction of remaining CPDs is indicative of slow repair). Using this analysis, our data shows that there was substantial CPD repair in WT cells within 1 h of irradiation (Fig. 4*A*). It is important to note that we used a relatively low UV dose (50 J/m^2^ UVC) in this study due to high UV sensitivity of the mutant strains. A clear repair strand bias was seen in the coding region in WT cells, with faster removal of CPDs from the TS relative to the NTS, consistent with published studies (14, 40). The remaining damage level was even lower 2 h post irradiation in the WT cells, with TS-biased repair still evident (Fig. S5*A*).

**Figure 4:**
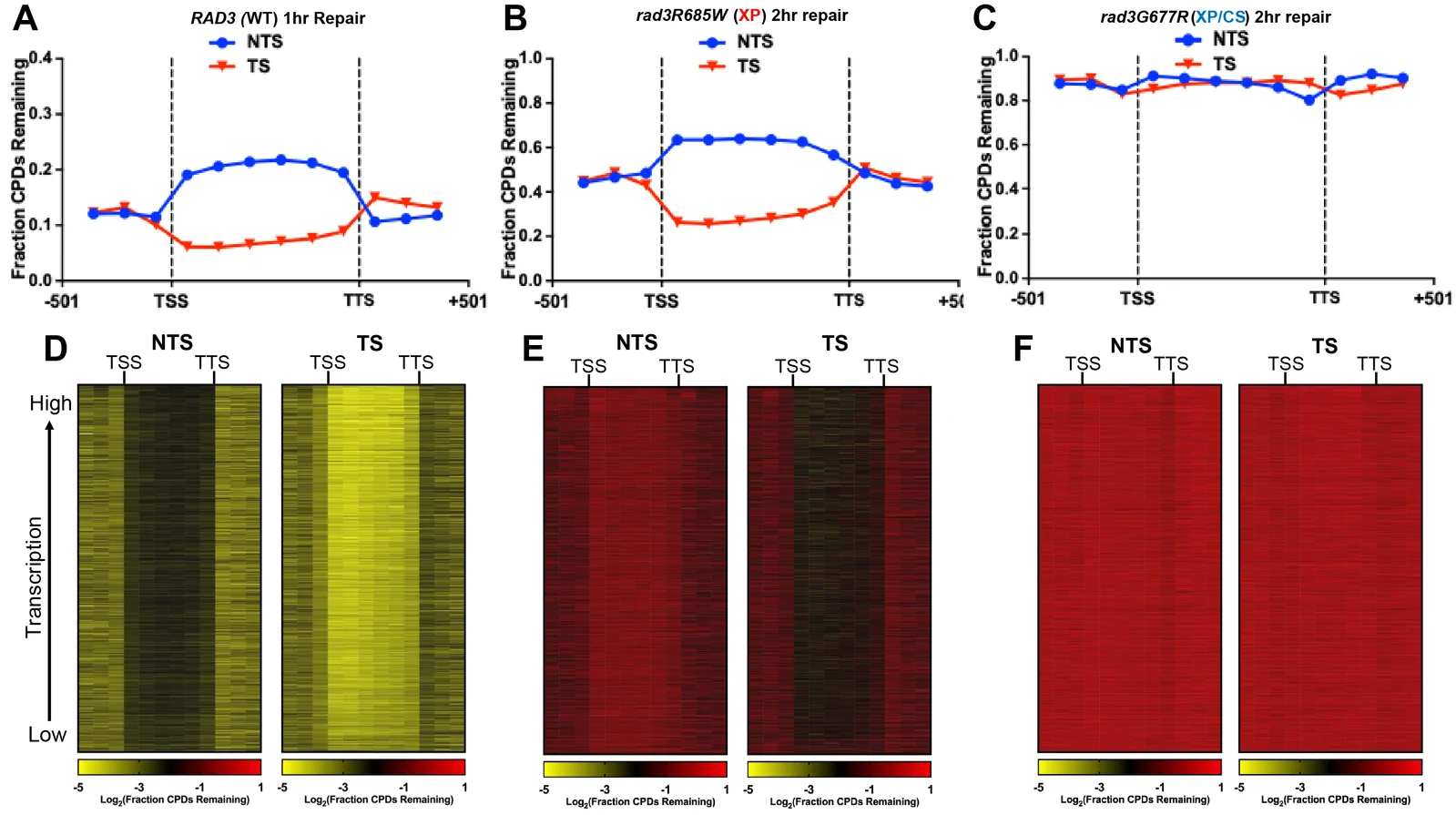
**CPD-seq 2.0 analysis of *rad3* mutants.***A,* plot showing average fraction of remaining CPDs on the NTS and TS for ∼5000 yeast genes in WT cells at 1 h. The gene coding region from TSS (transcription start site) to transcription termination site was split into six equal-sized bins and three additional bins upstream (*e.g.*, promoter) and downstream (terminator) were also included. CPD reads in each bin were counted and the number of CPDs at 1 h was normalized to that at 0 h (initial damage) to generate fraction of remaining CPDs. Each CPD-seq 2.0 experiment was performed twice and the second biological replicate data is shown in Supplemental Figures. *B* and *C,* same as *A*. Fraction of remaining CPDs was shown in *rad3R685W* and *rad3G677R* mutants at 2 h, to demonstrate repair on the NTS and TS. *D-F,* gene plot analysis of WT and *rad3* strains to examine the relationship of CPD repair to the level of transcription. The fraction of CPDs remaining on the TS and NTS in *(D)* (WT) and *(E* and *F)* (*rad3R685W*, *rad3G677R*) cells was shown in genes sorted by transcription frequency ordered from *low* to *high* (*e.g.*, *top* showing repair in actively transcribed genes). CPD, cyclobutane pyrimidine dimer; TS, transcribed strand.

Analysis of CPD repair in the two XP mutants revealed that they have slower repair than WT cells on both the NTS and TS, evidenced by the higher fraction of remaining CPDs 2 h after irradiation. However, repair is significantly faster on the TS than the NTS (Figs. 4*B*, S5*B* and S7). While this strand-biased repair is evident in the transcribed region between TSS and TTS, it is significantly diminished in the intergenic regions (*e.g.*, upstream of TSS or downstream of TTS), indicating that TC-NER is still active in the XP mutants. In contrast, analysis in the two XP/CS mutants shows that the small amount of repair that occurs does not exhibit a strand bias in coding regions (Figs. 4*C*, S5*C* and S7), indicating that both GG-NER and TC-NER are severely diminished.

In addition to the metagene repair analysis, which calculated the average repair rate of all genes, we also generated gene-by-gene repair heatmaps. In the heatmap analysis, yeast genes are sorted by their transcription frequency with highly expressed genes at the top, allowing us to visualize repair across individual genes. The repair heatmaps revealed wide-spread strand bias in WT and XP mutants, with highly expressed genes showing more significant strand differences than genes with lower expression levels (Fig. 4, *D* and *E*). We did not observe repair strand bias in XP/CS strains (Fig. 4*F*), even for highly expressed genes, indicating that TC-NER is abrogated.

To quantify the activity of TC-NER, we calculated the log ratio of remaining CPDs between the TS and NTS with a more negative log ratio indicative of more active TC-NER (45). As expected, the log_2_ TS/NTS ratio is negative in the WT and the two XP mutants (R685W and R603L). The value for R603L is less negative compared to R685W, suggesting the TC-NER activity in the R603L strain is lower compared with the R685W strain, consistent with the higher UV sensitivity in R603L cells (Fig. 2*A*). In contrast, the value was nearly zero in the two XP/CS mutants (Fig. S6), confirming the lack of TC-NER. Thus, while both GG- and TC-NER are reduced in the XP mutants, they do retain sufficient TC-NER activity to repair damage on the template strand of transcribed genes. In contrast, the XP/CS mutants exhibited a severe repair defect comparable to the Rad3 helicase-dead mutant as both GG- and TC-NER were abolished.

### High-resolution CPD analysis reveals partial repair in nucleosomes in XP mutants, but not in XP/CS mutants

Previous studies showed that repair of CPDs are significantly modulated by nucleosomes with the repair of CPDs near the dyad axis of a nucleosome much slower than that of CPDs in linker DNA (46, 47). We considered the possibilities that the reduced repair in XP mutants mainly impacts CPD lesions within the nucleosome rather than CPDs in both nucleosomal and linker DNA and that nucleosome positioning in the coding region may affect TC-NER in the mutant strains. To understand the effects of nucleosomes in different strains, we aligned the TSS of 5000 yeast genes and analyzed repair of CPD lesions at each nucleotide from −500 to +650 bp relative to the TSS, a region characterized by enrichment of strongly positioned nucleosomes near the 5′ end of yeast coding regions (Figs. 5 and S8). In accord with our previous results, periodic peaks of unrepaired CPDs occurred on the NTS strand, corresponding to nucleosomes positioned near the TSS with valleys of remaining CPDs in linker DNA between the nucleosomes of WT cells (Fig. 5*A*). As expected, repair on the TS is more even than on the NTS, with only slight variations between nucleosomal dyads and linker DNA, suggesting that nucleosomes have little effect on TC-NER in WT cells (14).

**Figure 5:**
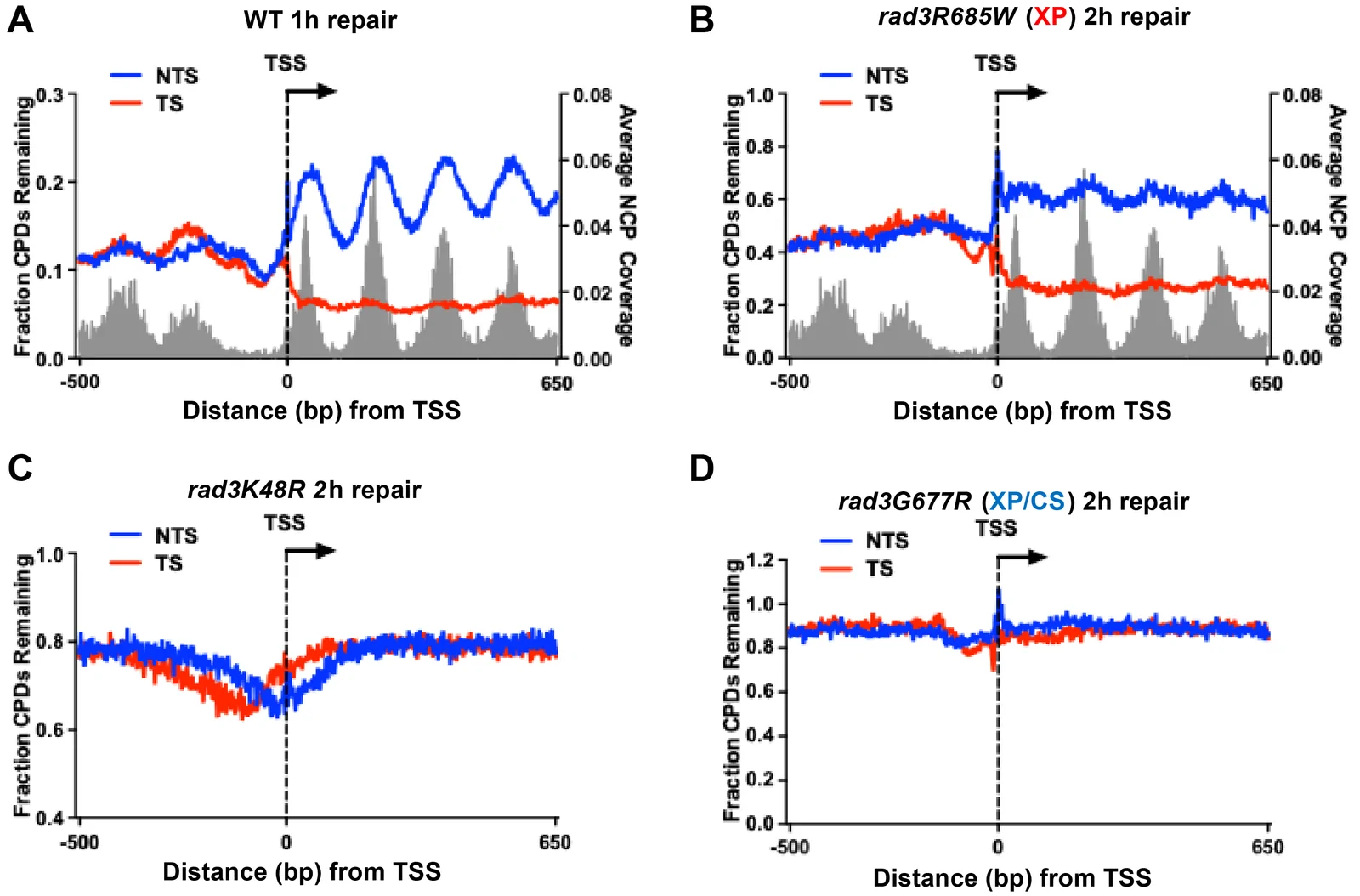
**High-resolution analysis of CPD repair in *rad3* mutants around the transcription start site.***A,* plot showing high-resolution repair analysis around the TSS at 1 h in WT cells. The analysis includes 500 bp upstream of the TSS (*e.g.*, promoters) and 650 bp downstream of the TSS in the coding region and shows remaining CPDs at each nucleotide. The *gray* background depicts nucleosomes positioned around the TSS using published nucleosomal dyad positions (58). *B–D,* similar to *A,* single-nucleotide resolution repair analysis around gene TSS in *rad3R685W*, *rad3K48R,* and *rad3G677R* mutant strains. CPD, cyclobutane pyrimidine dimer.

The pattern of CPD repair in the *R685W* XP mutant was somewhat similar to that seen in WT cells: fewer unrepaired CPDs were present on the TS while unrepaired CPDs were present in less pronounced periodic peaks along the NTS (Figs. 5*B* and S8*A*). The less strong periodic CPD pattern on the NTS suggests that the R685W mutation not only reduces GG-NER activity within the nucleosome, but also in the linker DNA between nucleosomes. In contrast, in the two *rad3* XP/CS mutants, similarly high levels of unrepaired CPD lesions were found along the NTS and TS both before and after the TSS, and the periodic pattern of GG-NER in coding region nucleosomes was absent, similar to the K48R mutant (Figs. 5, *C*, *D* and S8, *B*, *D*). These results are consistent with the lack of both GG-NER and TC-NER in the *rad3* XP/CS strains. The *rad3R603L* XP mutant had a phenotype that appeared intermediate between that of the *rad3R685W* XP mutant and the two *rad3* XP/CS mutants (Fig. S8*C*). While repair on the TS is still faster than the NTS, the periodic peaks of unrepaired CPDs were less prominent on the NTS, even when compared to *rad3R685W*. Thus, we conclude that while both GG-NER and TC-NER are more reduced in cells expressing the R603L compared with the other XP mutant, R685W, TC-NER is still more active in R603L compared with the adjacent G604D mutant (XP/CS).

### Analysis of phenotypes associated with patient derived human cell lines with XPD mutations

To confirm the results from the yeast studies indicating the XPD mutations resulting in both XP and CS cause a defect in TC-NER, we extended our analysis into patient-derived cell lines (38), examining their sensitivity to Illudin S, an agent that causes damage repaired by TC-NER (48, 49). We obtained XPD mutant cell lines established from XP (XB1R) and XP/CS (XP-CS2) individuals that express mutant versions of XPD, R683W and G602D, respectively, analogous to the yeast *rad3* mutants. We also included XP6BE, an XPD mutant cell line with one allele encoding the R683W mutant version and the other a p.Gly36-Arg61 deletion (50).The steady state expression levels of the mutant XPD proteins were reduced relative to the expression level in a comparable WT fibroblast cell line (Fig. 6*A*). Consistent with the yeast data, the XP-CS2 cells that expresses XPD-G602D from one allele, with the other allele not expressed, was significantly more sensitive to Illudin S than the WT cells (Fig. 6*B*), whereas the XB1R cells that are homozygous for the mutation encoding R683W exhibited slightly increased sensitivity compared to the WT cells. Surprisingly, the XP6BE cells that also express R683W are significantly more sensitive to Illudin S. A possible explanation is that, unlike the yeast *rad3* strain and the XB1R cells, the XP6BE cells also express a truncated version of XPD (50), which appears to impact TC-NER and may be responsible for the neurological problems of this individual (51).

**Figure 6:**
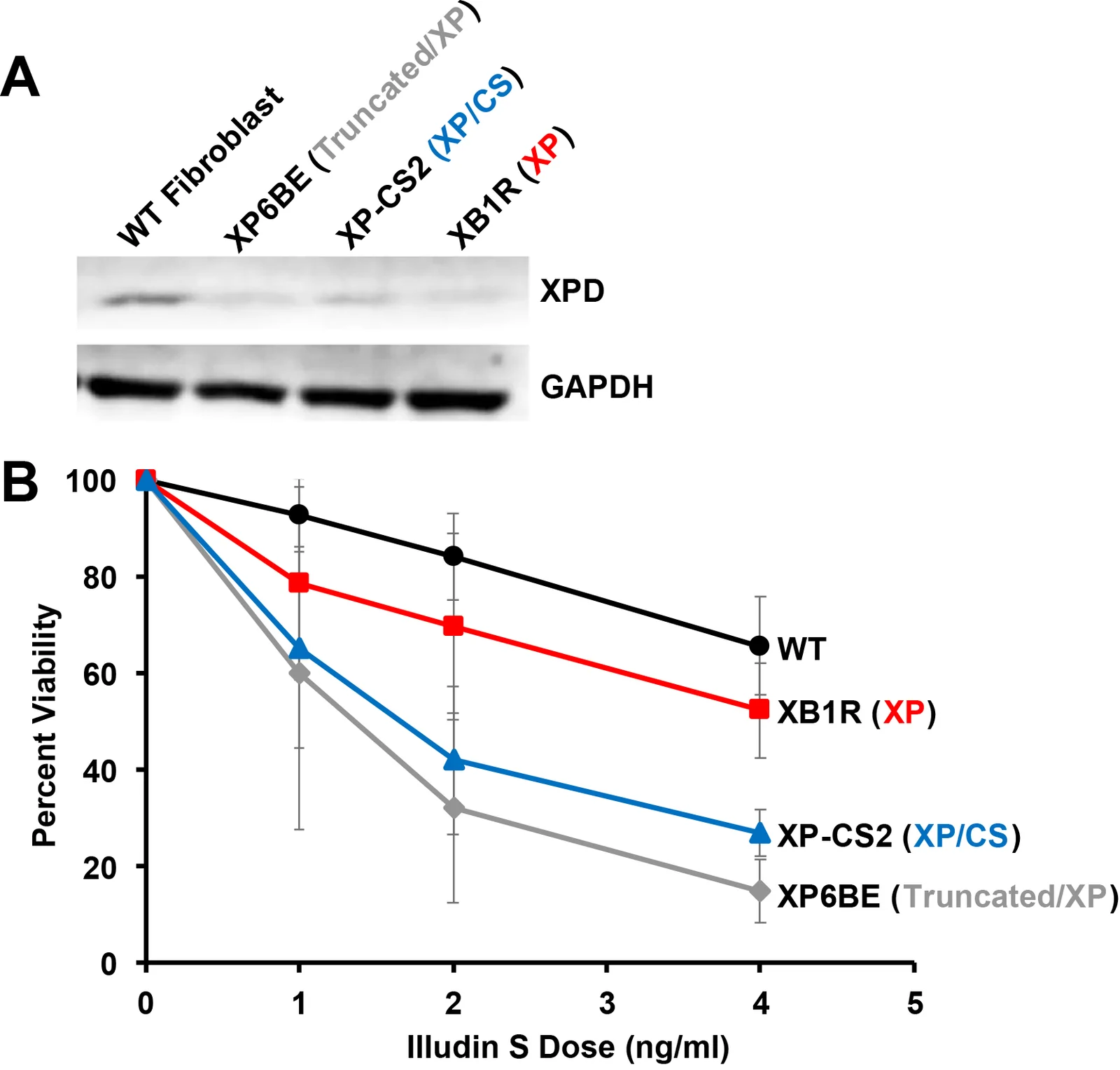
**Illudin Ssensitivity in patient derived XP and XP/CS human cells***A*, representative Western blot showing XPD protein in lysates prepared from different cell lines. *B*, sensitivity of XPD-WT and two XP-D patient derived fibroblasts, XB1R (XPD-R683W), XP-CS (XPD-G602D), and XP6BE (XPD-R683W and p.Gly36-Arg61 deletion), to varying doses of Illudin S (1 ng/mL, 2 ng/mL, 4 ng/mL). Percent viability was calculated by normalization of survived cells at each dose to a no drug control. The MTT assays were performed on four biological replicates and three technical replicates, the data were averaged, and standard deviations were calculated.

## Discussion

Germline mutations in the human *XPD* gene have been identified as the causative factor in two inherited genetic disorders, TTD and XP, as well as a combination of XP with a third genetic disorder CS (3, 36). TTD is considered a transcription disorder (35, 38, 52, 53), while XP and CS phenotypes are caused by defects in NER (54). Notably, CS is characterized at the cellular level by a defect in the TC-NER subpathway of NER (6). XPD is an essential factor in both transcription initiation and NER, functioning as a subunit of the TFIIH complex (21, 25). While the initial DNA damage recognition steps of the GG- and TC-NER subpathways are different, the subsequent steps involving TFIIH appear to be shared by the two pathways (16, 23, 27). Despite extensive structural and biochemical studies of XPD, it is still not known how different amino acid changes in XPD, some of which are close together, can cause either XP or XP/CS. Notably, the effects of amino acid changes causing XP or XP/CS on the relative activity of the two NER subpathways, has not been examined in detail.

In this study, we have used budding yeast to model the repair defects in XP and XP/CS, taking advantage of the highly conserved protein sequence between yeast Rad3 and human XPD. We chose two pairs of closely located amino acid changes that cause either XP (R683W, R601L) or XP/CS (G675R, G602D), and introduced point mutations into the *RAD3* gene using CRISPR-Cas9 to generate the same changes at the conserved residues in Rad3. While the effects of these amino changes on the ssDNA binding, ATPase and helicase activities of an archaea XPD homolog have been determined (31, 32, 55), these studies did not identify a specific biochemical defect associated with either XP or XP/CS. Strikingly, the yeast *rad3* strains modeling XP/CS were much more sensitive to UV irradiation and had a much lower rate of CPD removal compared with yeast *rad3* strains modeling XP. Indeed, the phenotype of the *rad3* XP/CS strains resembled that of the K48R strain that expresses a version of Rad3 that lacks ATPase and helicase activities. Interestingly, there was a modest difference in the DNA repair deficiency between the two yeast strains modeling XP with the R603L strain being slightly more UV sensitive than the R685W. This would not have been predicted from the biochemical data as the archaea R683W homolog has more severe defects in ssDNA binding, helicase and ATPase activity compared with the archaea R601L homolog (32); however, the helicase activity of both mutants was similarly decreased, with the R683W homolog being the most reduced. This biochemical data for these particular mutants needs further investigation as the archaea species has a different number of amino acids and some function may not be retained in full when compared to the human XPD.

To investigate the effect of XPD mutations on repair in more depth, we used our published CPD-seq 2.0 method to determine TC-NER and GG-NER activities in yeast strains carrying XP or XP/CS mutations. As expected, we observed enhanced repair of the TS compared with the NTS in WT yeast cells, reflecting TC-NER activity. Asymmetry of strand repair was evident in the XP mutant strains, albeit to a lesser extent than in WT cells whereas no strand repair asymmetry was detected in the XP/CS strains (Figs. 4 and S6). Thus, our results utilizing a genome-wide strand specific repair assay provide compelling evidence supporting the earlier studies (18) suggesting that certain amino acid changes in XPD cause defects in both GG- and TC-NER and it is the defect in TC-NER that likely underlies the development of CS, in addition to XP. In addition, our results show that while the XP mutants had defects in GG-NER, they retained TC-NER activity. The remaining TC-NER activity in the two XP mutants is consistent with mild UV sensitivity in yeast cells and lack of neurodegeneration symptoms (CS) in the corresponding human patients.

In summary, we have used yeast as a model to demonstrate that mutations in the XPD gene that cause a combination of XP and CS (XP/CS) have a much more severe defect in NER, as well as a specific defect in TC-NER, compared with mutations in XPD that cause XP alone. To further support this conclusion, we examined sensitivity to Illudin S, an agent causing damage repaired by TC-NER, in patient-derived cell lines expressing the mutant versions of XPD analyzed in the yeast studies. As expected, a representative XPD XP/CS cell line XP-CS2 (G602D), was sensitive to Illudin S whereas a representative XPD XP cell line XB1R that is homozygous for the mutation encoding XPD R683W, was not. However, another XPD XP cell line, XP6BE, that also expresses XPD R683W was sensitive to Illudin S. Notably, although the mutation resulting in the expression of XPD R683W is the most frequently observed mutation in *XPD* (36), and assumed to be responsible for the cancer predisposition that is the hallmark of XP, there is evidence that the second XPD allele in heterozygotes contributes to the presence and severity of other clinical symptoms such as neurodegeneration in addition to skin cancer. For example, the individual from whom the XP6BE cell line was established had severe neurological symptoms in addition to skin cancer (51). Thus, our studies suggest that truncated version of XPD encoded by the second allele in XPBE cells negatively impacts TC-NER that in turn contributes to neurodegeneration. Furthermore, while our studies do indicate that there are differential requirements for XPD function in GG- and TC-NER, they have limitations in revealing mechanistic insights into the differences. Another limitation is our data is mainly generated in yeast with a focus on NER. Previous studies suggest that defects in other repair pathways such as base excision repair, gene transcription, and mitochondria function, are also linked with CS in humans (56). Further studies are needed to understand the complex mechanisms underlying human XP and XP/CS phenotypes. As both GG- and TC-NER rely on TFIIH for DNA repair, it remains unclear why some mutations in XPD selectively affect GG-NER while a few mutations disrupt both subpathways. We speculate that the pre-existing bubble generated by RNAP II may reduce the dependence on XPD’s helicase and/or ATPase activity for DNA unwinding (Fig. S9), thereby explaining why TC-NER is retained in XP-causing mutants. On the other hand, GG-NER is initiated without a transcription bubble and the almost fully annealed DNA duplex likely requires a strong TFIIH activity to initiate *de novo* DNA unwinding. Consequently, XPD mutations affecting its enzymatic activity may have a more pronounced impact on the GG-NER subpathway than on TC-NER. The development of genome-wide assays, such as CPD-seq, to measure strand specific repair in patient-derived cell lines will likely provide more insights as to how defects in GG- and TC-NER contribute to cancer predisposition, growth and developmental delays and neurodegeneration in individuals with XP and a combination of XP and CS.

## Experimental procedures

### Yeast strains

All yeast strains were constructed in the *MATa leu2Δ0 met15Δ0 ura3Δ0 trp1Δ0* background. Genotype and strain table (Table S2).

### CRISPR-Cas9 gene editing

We engineered five point mutations, including one helicase-dead and two pairs of XP and XP/CS, into the yeast RAD3 gene using CRISPR-Cas9 gene editing (42). The WT yeast strain, MP019, was first transformed with a Cas9 expressing plasmid, pTO22. DNA oligos expressing specific guide RNAs targeting the editing sites were cloned into a second plasmid, pTO40. The guide RNA expressing plasmid, together with single-stranded donor DNA containing the designed mutations, were subsequently transformed into MP019 carrying pTO22. The pTO22 and pTO40 plasmids carry Leu2 and Ura3 selective markers, respectively. The transformed cells were selected on selective plates lacking Leucine and Uracil to identify potentially edited clones. Three individual clones from each transformation plate were collected for Sanger sequencing to confirm point mutation at the designed site in the donor DNA. After Sanger sequencing confirmation, yeast cells were grown in rich media to remove both plasmids.

### UV sensitivity assay

Yeast cells were grown at 30 °C in YPD medium (ref) to mid-log phase (∼0.8 OD600). For spotting assays, strains were serially diluted in fresh YPD medium and spotted in 10-fold serial dilutions onto YPD plates. Plates were exposed to different doses of UV-C light (254 nm), and incubated in the dark at 30 °C for 2 to 3 days. Images were taken on an Azure Biosystems 200 machine.

### Alkaline gels to measure CPD repair

Yeast cells were grown at 30 °C in YPD medium to mid-log phase (∼0.8). Cells were pelleted, resuspended in ddH_2_O, and irradiated with 50 J/m^2^. Following UV treatment, cells were pelleted, resuspended in YPD, and incubated at 30 °C in the dark. Cells were collected immediately (0 h) and at 1 h, 2 h, and 3 h after UV irradiation. Cells from each timepoint were pelleted and stored at −80 °C. gDNA was extracted from samples (43) and treated with or without T4 Endonuclease V (NEB, M0308) for approximately 2 h at 37 °C. The DNA samples were run on a ∼1.2% agarose/10% alkaline gel at 27-28V overnight for ∼18 h at 4 °C. After electrophoresis, the gel was rinsed with ddH_2_O, the pH was neutralized, and the gel was stained with SYBR Gold in TAE buffer for at least 1 h. The gel was destained in ddH_2_O for ∼ 1 h, and then scanned using a Typhoon FLA 7000 imager. The amount of DNA damage was quantified with ImageQuant software by analyzing the pixel density of each lane. Once the density is calculated, the midpoint of each timepoint can be determined, using the DNA ladder as a reference. Midpoints of each smear can be compared to quantify the amount of CPDs retained or repaired. Biological replicates were analyzed for each strain.

### CPD-seq 2.0 library preparation and sequencing

Yeast cells were grown at 30 °C in YPD media to mid-log phase (∼0.8). Cells were pelleted, resuspended in ddH_2_O, and irradiated with 50 J/m^2^ UVC. Immediately following UV irradiation, cells were pelleted, resuspended in fresh YPD, and incubated at 30 °C in the dark. Cells were collected immediately after UV irradiation (0 h) and at 1 h or 2 h after repair was initiated, then pelleted and stored at −80 °C. After isolation of gDNA, 5 ug, CPD-seq libraries were prepared as previously described (14, 39). The quality of library was evaluated by conducting a check PCR using primers complementary to two adapters. A significant enrichment of PCR signal in UV-irradiated cells over the No UV control is required before sending samples for Illumina sequencing. Two biological replicates were analyzed for each CPD-seq 2.0 experiment.

### Data processing and analysis

The raw CPD-seq 2.0 data were demultiplexed using barcode information in each sample. Adaptors sequences were removed using trimmomatic (57) and the paired-end reads were aligned to the yeast reference genome (sacCer3) using Bowtie2. The alignment results were converted to BAM files using SAMtools and PCR duplicates were removed using Picard MarkDuplicates function. The forward read (Read1) was extracted from the cleaned BAM file and the dinucleotide associated with each Read1 was retrieved using BEDTools. Reads that were associated with dipyrimidines (*e.g.*, TT, TC, CT, and CC) were retrieved (CPD reads) for CPD repair analysis. Reads that were not associated with dipyrimidines (only account for a small fraction of UV-damage samples) were considered background and excluded from downstream analysis.

Repair analysis in yeast genes was performed using custom Python scripts. For analysis shown in Figure 4, genes were first binned and the number of CPD reads in each bin was counted at 0 h (initial) and different repair time points (1 or 2 h). The number of CPD reads after repair was normalized by the read count at 0 h, which results in the fraction of remaining CPDs. As the fraction of remaining CPDs can be affected by different sequencing depths, we used the overall percent of remaining CPDs generated in Figure 2*E* to obtain a scaling factor for each CPD-seq experiment. The corrected fraction of CPD remaining by the scaling factor is plotted using GraphPad. For analysis shown in Figure 5, the number of CPD reads was counted at each nucleotide, from −500 to +650 bp, for ∼5000 yeast genes. The CPD counts after repair was normalized to the initial CPD counts. The resulting fraction of remaining CPDs was adjusted by the scaling factor to generate the plots.

### Western blotting

Yeast cells were grown in YPD at 30 °C to late log phase and whole cell extracts were prepared using the TCA procedure (ref). Thirty-5 μg of protein were loaded on 10% SDS-PAGE gels. Western blot analysis was performed with antibodies against yeast Rad3 (Invitrogen, PA5-32174) and HRP-GAPDH (Cell Signaling, D16H11). Blots were developed after exposure to X-ray film or scanned on a Typhoon FLA 7000 imager. Two biological replicates were examined.

### Human cell lines and cell culturing

Patient derived human cell lines XB1R (GM03615), XP-CS2 (GM03248), and XP6BE (GM08207), were purchased from Coriell (Table S3). These cells and a WT fibroblast immortalized cell line, provided by the Dr Hua-ying Fan lab, were grown in Dulbecco’s Modified Eagle Medium + Glucose and L-Glutamine (DMEM, Gibco 11965-092) supplemented with 15% Fetal Bovine Serum (Sigma-Aldrich F0926) at 37 °C using a humidified 5% CO_2_ incubator. Cells were routinely grown until passage 14. Cells were washed with 1X PBS, followed by the addition of trypsin (Thermo Fisher Scientific, 25200056) to remove the cells from the plate in order to maintain them and perform assays. No antibiotics were used during cell culturing, and *Mycoplasma* detection was performed routinely.

Stocks were made for each cell line by addition of 10% DMSO to culture medium, followed by freezing at −80 °C and storage in liquid nitrogen.

### Measurement of sensitivity to illudin S

Cells were seeded onto 96 well plates at ∼5000 cells/well in a volume of 100 μl and cultured for 24 h. Illudin S (Cayman Chemical, 17451) was serially diluted in PBS, added to fresh media and pipetted into wells after removal of the plating media. Plates were then incubated at 37 °C for 72 h. 10 microliters of MTT substrate (Cell Proliferation Kit I, Roche, cat. #11465007001) were added to each well and incubated for 4 h at 37 °C. One hundred microliters of solubilization buffer was added to lyse cells and incubation was continued overnight at 37 °C. Plates were scanned with a Clario Star plate reader at 570 nm and 690 nm wavelengths. All experiments were repeated in four biological and 2 to 3 technical replicates.

To quantify cell viability, the 690 nm reading was subtracted from the 570 nm reading to remove background. These values can then be averaged together per Illudin S dose, and normalized to the no drug wells. This can then be analyzed as percent viability and a function of Illudin S sensitivity.

## Data availability

CPD-seq 2.0 data generated in this study is deposited to the Gene Expression Omnibus (GEO) database under accession GSE334098. The custom CPD-seq 2.0 analysis code is deposited to Zenodo (https://zenodo.org/records/21876170).

## Supporting information

This article contains supporting information.

## Conflict of interest

A. H., A. E. T., and P. M. declare that they have no conflicts of interest with the contents of this article. A. E. T. is an Editorial Board Member/Editor-in-Chief/Associate Editor/Guest Editor for this journal and was not involved in the editorial review or the decision to publish this article.
