# Supplementary material for: UV damage mapping reveals different impacts of yeast XPD mutations on global genomic and transcription-coupled DNA repair
Source: J Biol Chem. 2026 Aug 25;302(10):113483. doi: 10.1016/j.jbc.2026.113483 (PMC13627133; doi:10.1016/j.jbc.2026.113483)
Supplement: Supplementary file 1 [file mmc1.pdf]

Supplementary Information

Mutation	Symptom	Domain
G47R	XP/CS	Helicase Motif I
T76A	XP	Helicase Motif Ia
D234N	XP	Helicase Motif II
R511Q	XP	Helicase Domain II
S541R	XP	Helicase Motif IV
Y542C	XP	Helicase Motif IV
R601L	XP	Helicase Motif V
R601W	XP	Helicase Motif V
G602D	XP/CS	Helicase Motif V
R666W	XP/CS	Helicase Motif VI
G675R	XP/CS	p44 Interacting Region
D681N	XP	p44 Interacting Region
R683W	XP	p44 Interacting Region
R683Q	XP	p44 Interacting Domain
Q726am	XP	p44 Interacting Domain

Table S1. XP and XP/CS mutations in human XPD, the disorders associated with the mutations, and the domain where each mutation is located.

Strain	Genotype	Source
MP019 (WT)	*MATa leu2Δ0 met15Δ0 ura3Δ0 trp1Δ0*	Ref 14
MP084	*MATa leu2Δ0 met15Δ0 ura3Δ0 trp1Δ0 rad3K48R*	Ref 14
YMB006	*MATa leu2Δ0 met15Δ0 ura3Δ0 trp1Δ0 rad3R685W*	This study
YMB002	*MATa leu2Δ0 met15Δ0 ura3Δ0 trp1Δ0 rad3G677R*	This study
YMT003	*MATa leu2Δ0 met15Δ0 ura3Δ0 trp1Δ0 rad3R603L*	This study
YMB004	*MATa leu2Δ0 met15Δ0 ura3Δ0 trp1Δ0 rad3G604D*	This study

Table S2. *S. cerevisiae* strains

Cell Line	Genotype	Source
XP6BE	Allele 1: XPD-R683W<br>Allele 2: Truncated in the first XPD helicase motif	Coriell (cat # GM08207)
XB1R	Allele 1: XPD-R683W<br>Allele 2: XPD-R683W	Coriell (cat # GM03615)
XP-CS2	Allele 1: XPD-G602D<br>Allele 2: Not expressed	Coriell (cat # GM03248)
CSB WT	WT fibroblast cell line	Fan Lab

Table S3. Human cells

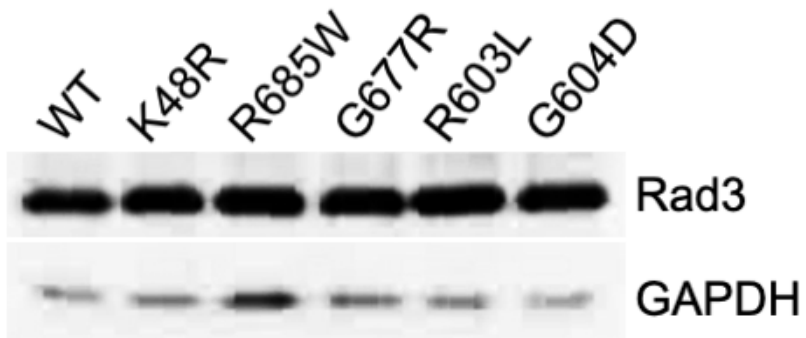

Figure S1. Representative western blot of Rad3 protein in *rad3* XP and XP/CS mutants. TCA lysates were prepared from cells grown in YPD to mid-log phase, and 35 micrograms were loaded onto SDS-PAGE gels. Blots were incubated with antibody against yeast Rad3, and HRP-GAPDH was used as a loading control.

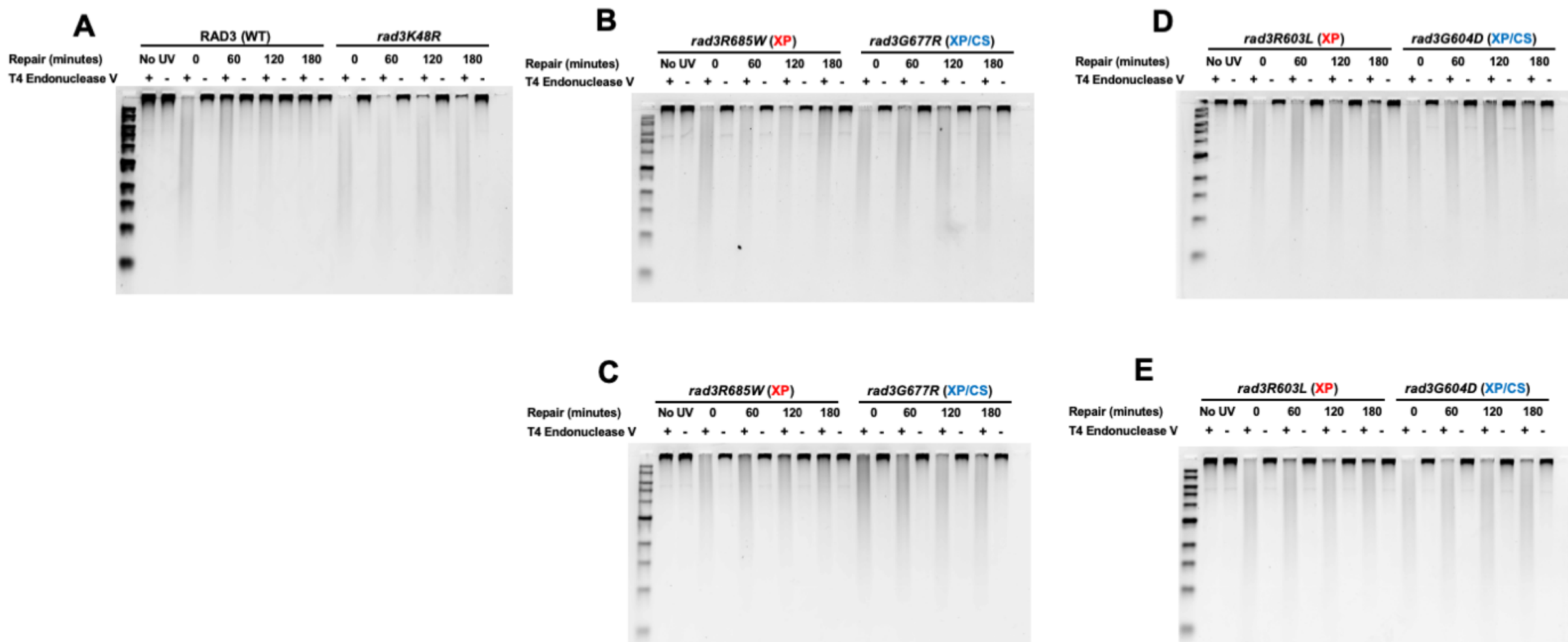

Figure S2. Replicates of denaturing alkaline gels after UV treatment. **A** (WT, *rad3K48R)*; **B, C** (*rad3R685W*, *rad3G677R*); **D, E** (*rad3R603L*, *rad3G604D*).

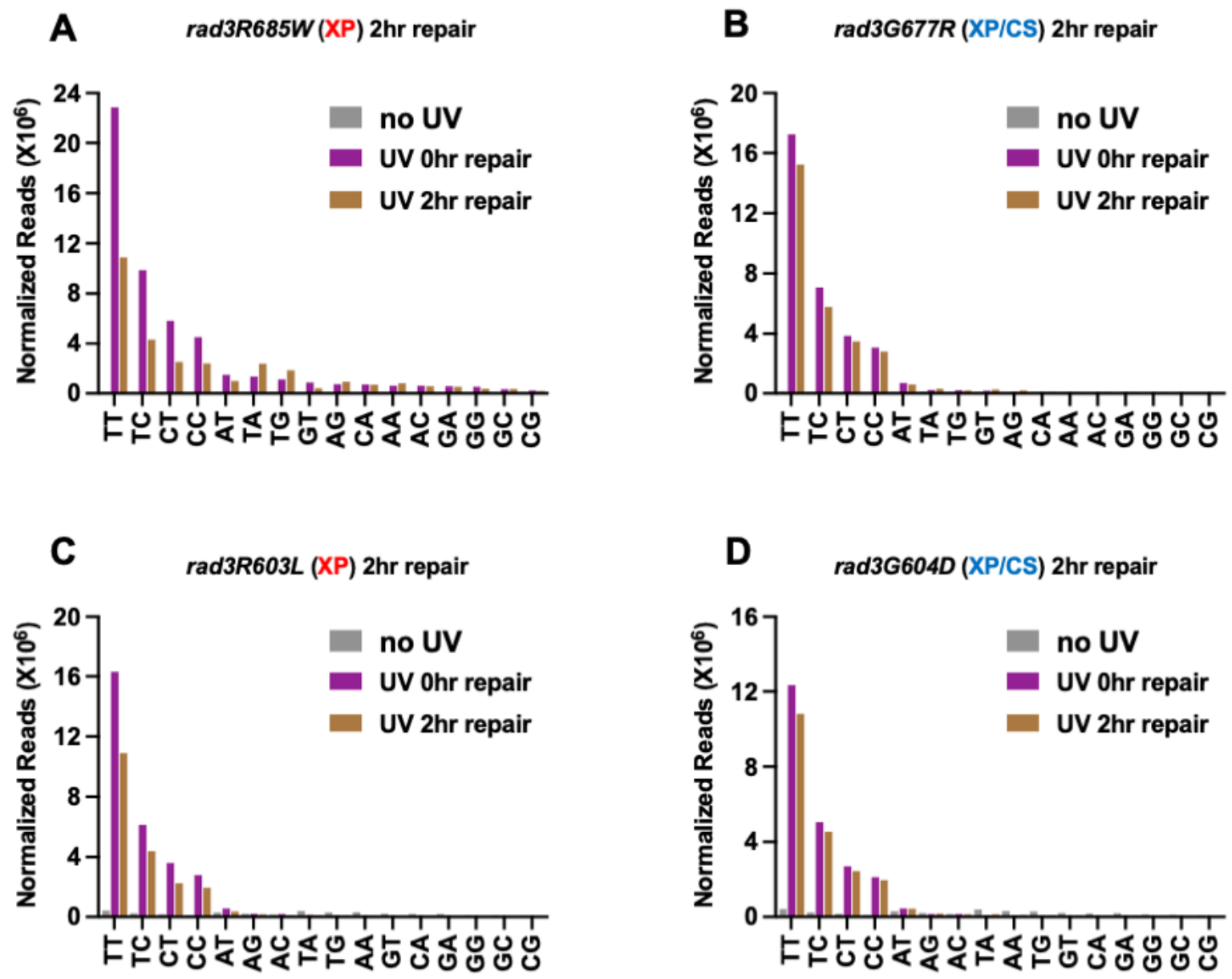

Figure S3. Normalized CPD-seq reads after UV irradiation.
Enrichment of CPD reads (e.g., TT, TC, CT, and CC) after 50J/m^2 UV irradiation in each mutant strain. The read counts after repair at 2hr are normalized by the scaling factor generated by the alkaline gel analysis to correct for sequencing depth difference between 0hr and 2hr.

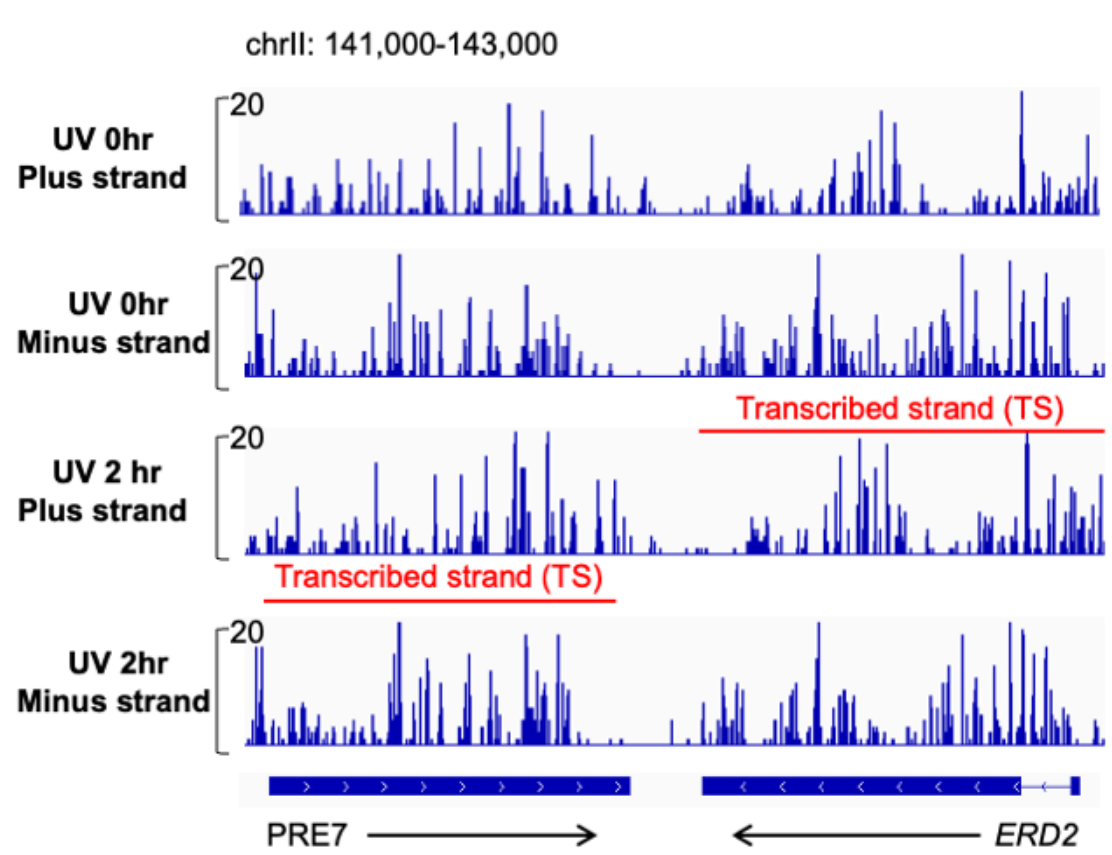

Figure S4. Lack of NER activity in helicase dead mutant with CPD-seq analysis
Distribution of CPD reads at 0hr and 2hr of repair for *PRE7* and *ERD2* genes in yeast helicase dead mutant (*rad3K48R*). The red line indicates the transcribed strand, and the y axis depicts the amount of CPD-seq reads. Gene coordinates and image drawn using the Integrative Genomics Viewer.

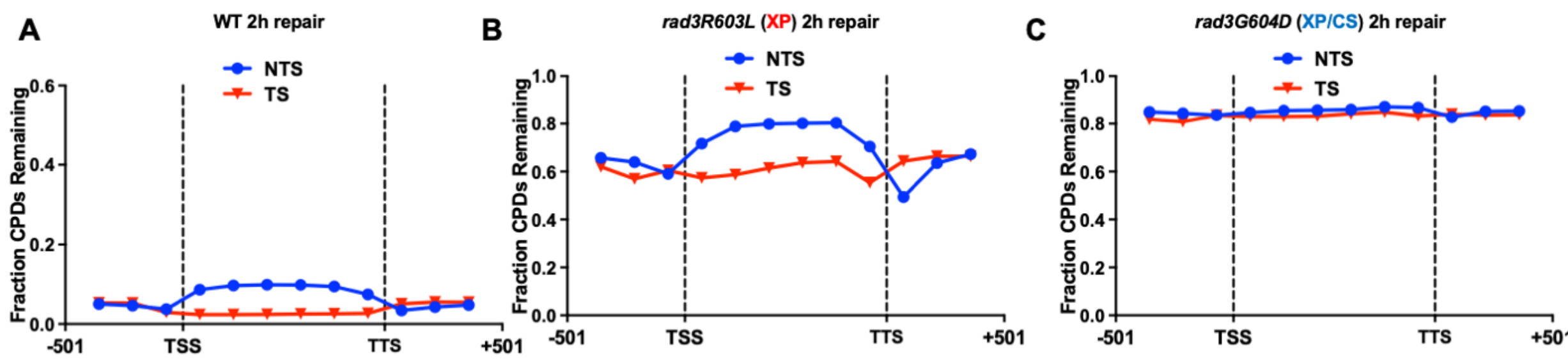

Figure S5. CPD-seq analysis of WT, *rad3R603L and rad3G604D* mutants.
CPD-seq data for WT-2hr (**A**), *rad3R603L* (XP) (**B**) and *rad3G604D* (XP/CS) (**C**) mutants. The average fraction of CPDs remaining after 2hr of repair was plotted for both the transcribed strand (TS) and non-transcribed strand (NTS) of approximately 5000 yeast genes. The number of unrepaired CPDs remaining after 2hr was normalized to the initial number of CPDs formed at 0hr to calculate the fraction of CPDs remaining on each strand. CPD-seq data were divided into 6 equally sized bins spanning the TSS to TTS and 3 bins representing the 5’ and 3’ flanking regions, and the fraction of CPDs remaining was analyzed in each bin. CPD-seq data were scaled using the overall repair data for the 2hr time point from alkaline gel analysis (**Fig 2E**).

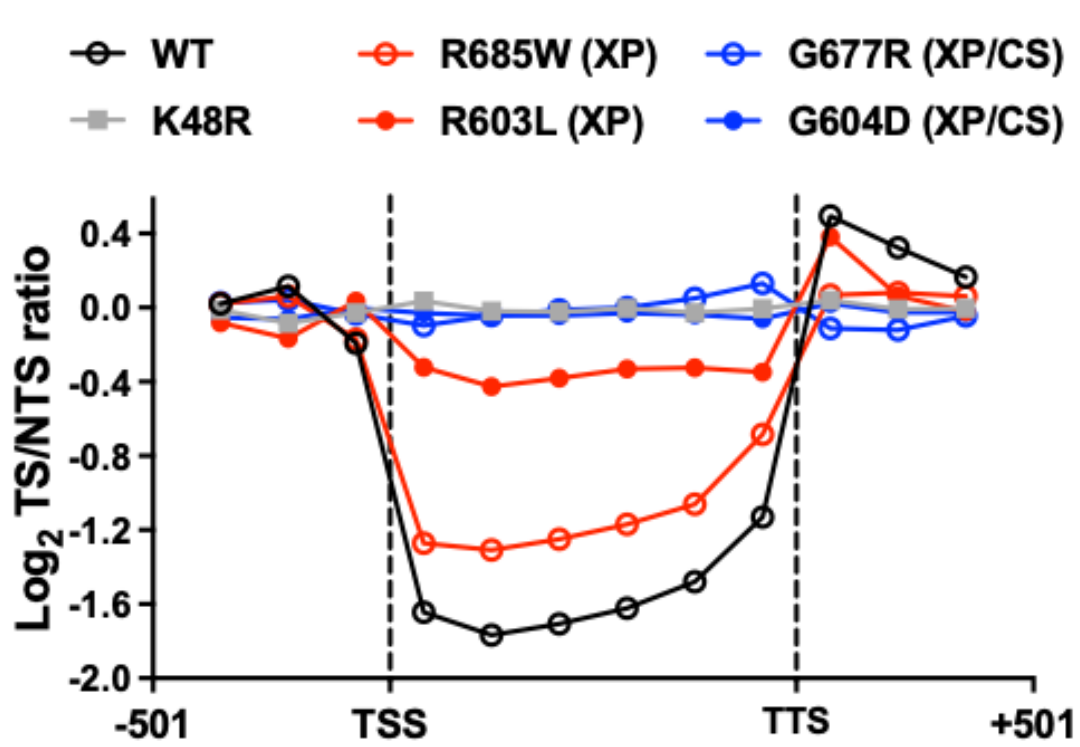

Figure S6. Log$_2$ Ratio of CPDs remaining between TS and NTS (TS/NTS)
Plot showing the log$_2$ ratio of unrepaired CPDs on the TS relative to the NTS in each bin (see Methods) in WT and rad3 mutants. The more negative value indicates more active TC-NER activity (e.g., less remaining CPDs on the TS). Lack of TC-NER repair in XP/CS and K48R mutant strains is indicated by the log$_2$ ratio at zero.

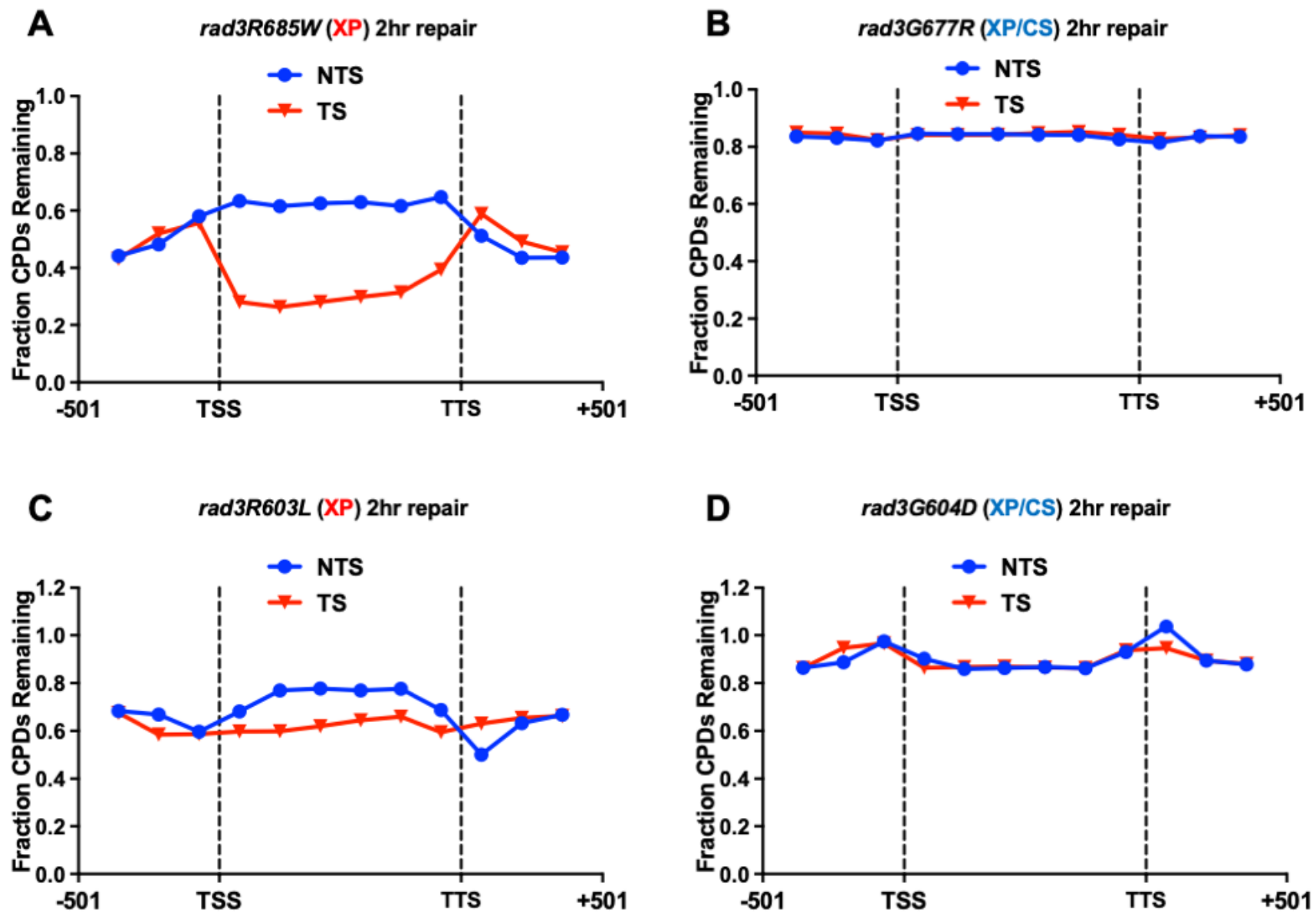

Figure S7. Independent repeat of CPD-seq analysis of *rad3* XP and XP/CS mutants.

Bin plot analysis of the average fraction of CPDs remaining after 2 hours of repair was plotted from the CPD-seq data along the transcribed strand (TS) and non-transcribed strand (NTS) of ~5000 yeast genes in (**A**, **C**) *rad3R685W* and *rad3R603L* XP cells and *rad3G677R* (**B**, **D**) *rad3D604D* XP/CS cells.

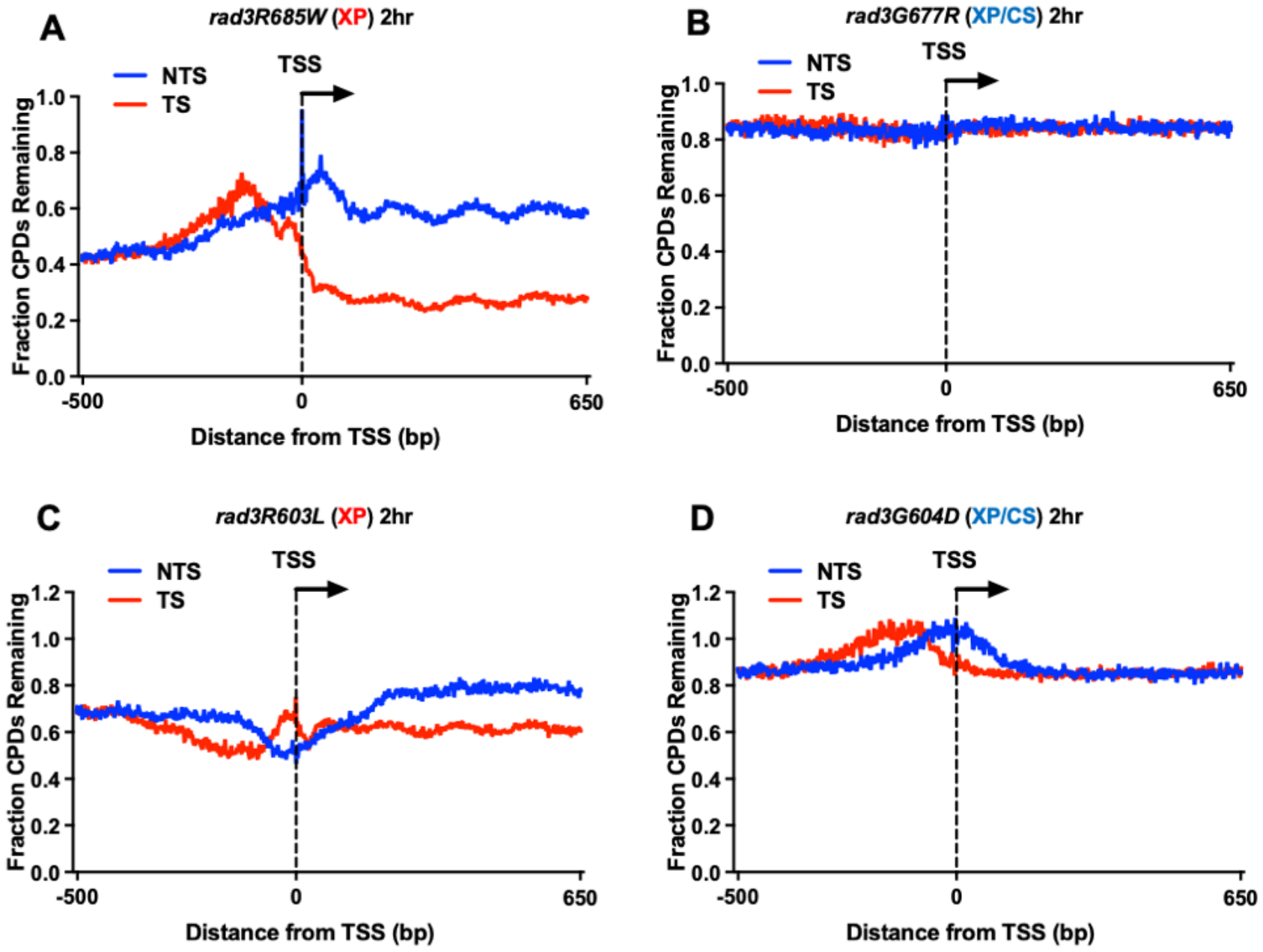

Figure S8. Independent replicate of high-resolution analysis of CPD-seq data in *rad3* XP and XP/CS mutants. Data was generated in independent CPD-seq 2.0 experiments performed in XP (**A** and **C**) and XP/CS (**B** and **D**) strains.

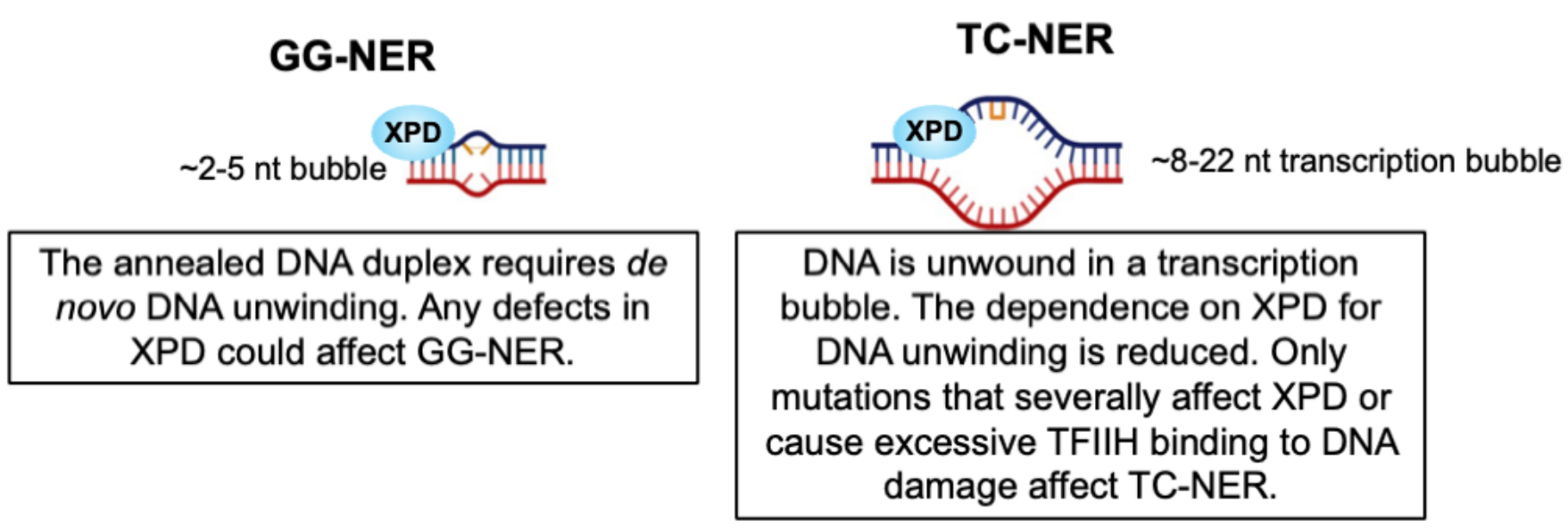

Figure S9. A working model for different impacts of XPD mutations on GG- and TC-NER.
